# Supplementary material for: CODC: a Copula-based model to identify differential coexpression
Source: NPJ Syst Biol Appl. 2020 Jun 19;6:20. doi: 10.1038/s41540-020-0137-9 (PMC7305108; doi:10.1038/s41540-020-0137-9)
Supplement: Supplementary file 2 — Supplementary figure-1 [file 41540_2020_137_MOESM2_ESM.pdf]

Supplementary Figure-1

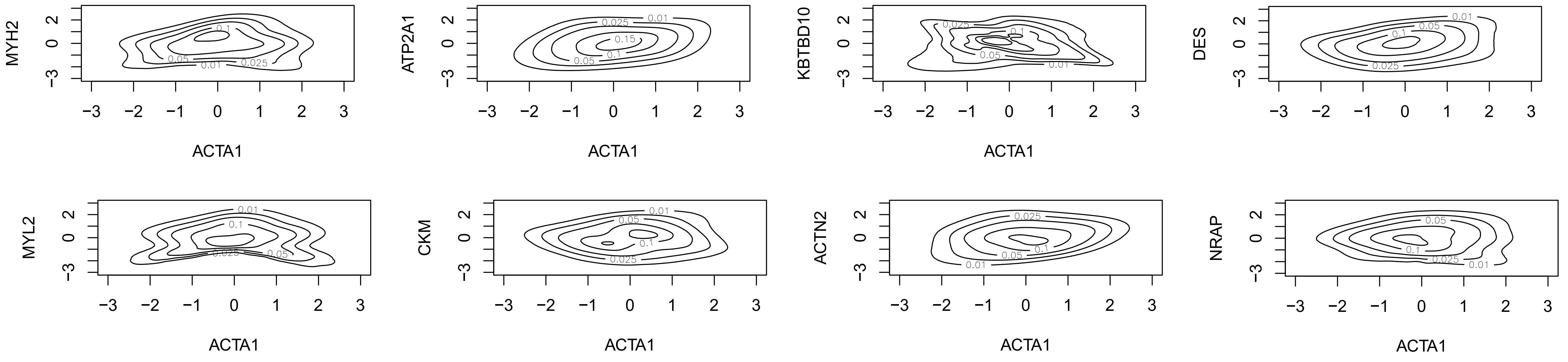

Figure shows some marginal normal contour plots of copula density for some of the gene pairs during estimation in BRCA Dataset

Supplementary Figure-2

I. RESULTS

A. Supplementary figures for BRCA data

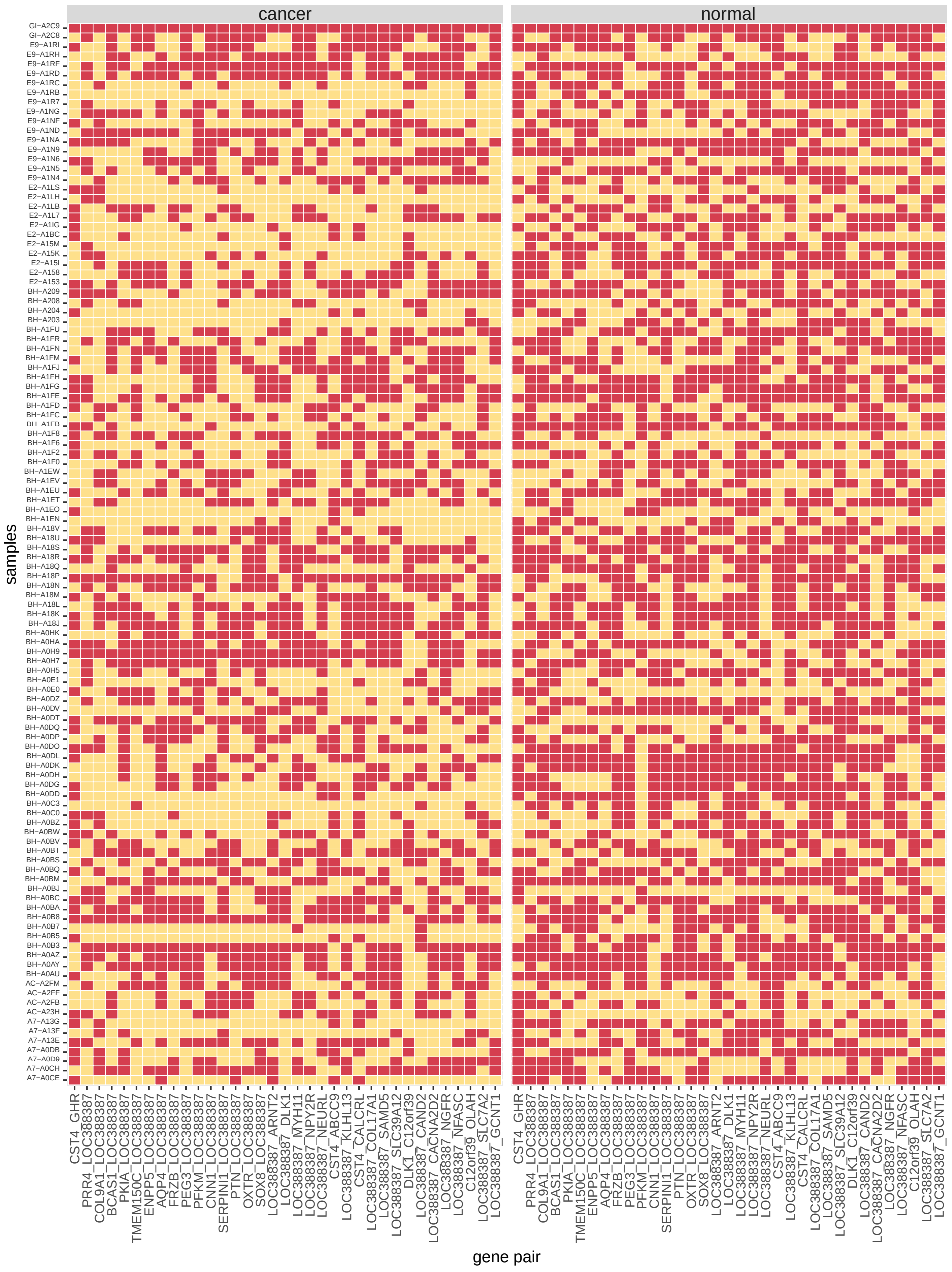

Fig. 1. Figure shows a heatmap representation of binary matrix constructed from the expression matrix of top differentially coexpressed gene pairs in BRCA data. Expression values of a gene pair showing the same pattern is indicated as '1' and showing a different pattern is indicated as '0' in the matrix. The columns representing differentially coexpressed gene pairs which show positive correlations normal stage while show negative correlation in cancer stage.

Supplementary figures for LIHC data

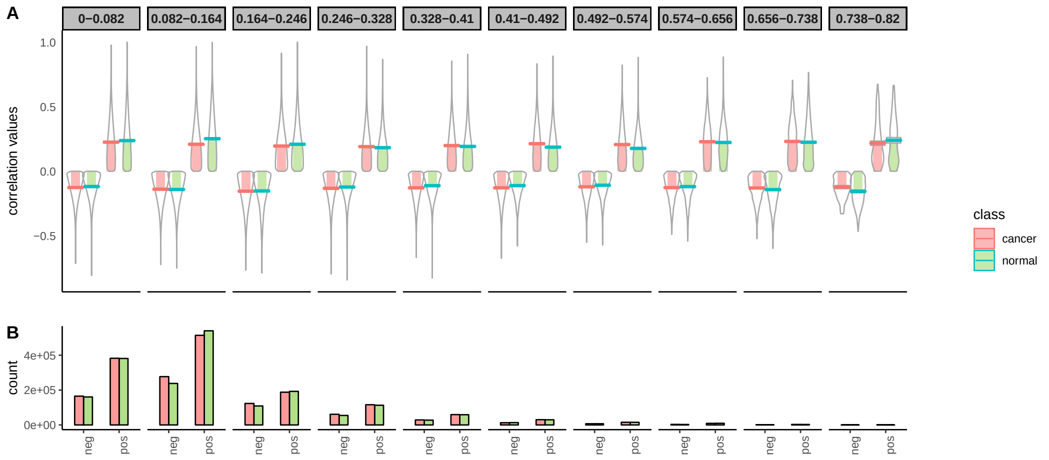

Fig. 1. Figure shows distribution of correlation values in normal and cancer samples of LIHC data with the DC_Copula score. Panel-A shows the distribution for different DC_Copula scores. Here, 4 pirate plots are shown in each facet, two for positive and two for negative correlations. The violins in each facet represents the distribution of positive and negative correlations of gene pair in normal and cancer samples. Panel-B shows a bar plot representing the number of positive and negatively correlated gene pairs in normal and cancer samples in each facet

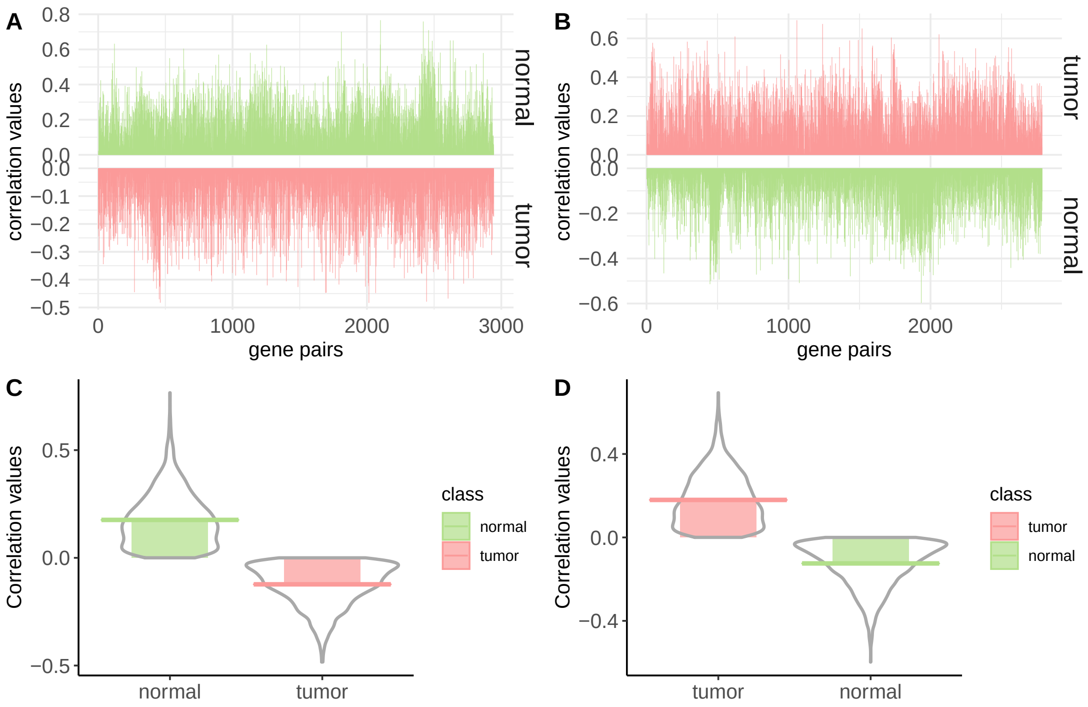

Fig. 2. Figure shows visualizations of gene pairs having DC_copula score greater than 0.56. Panel-A and Panel-B shows visualization of correlation values of gene pair having positive correlation in normal and negative correlation in tumor and vice-versa, respectively. Panel-C and Panel-D represents the distribution of correlation values according to panel-A and panel-B respectively.

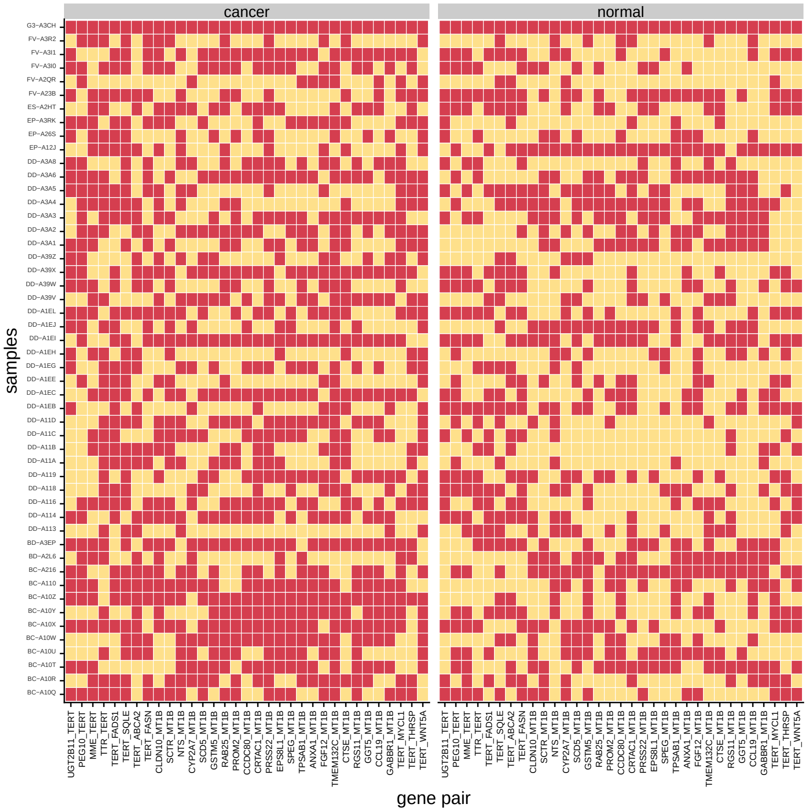

Fig. 3. Figure shows a heatmap representation of binary matrix constructed from the expression matrix of top differentially coexpressed gene pairs in LIC data. Expression values of a gene pair showing the same pattern is indicated as '1' and showing a different pattern is indicated as '0' in the matrix. The columns representing differentially coexpressed gene pairs which show positive correlations cancer stage while show negative correlation in normal stage.

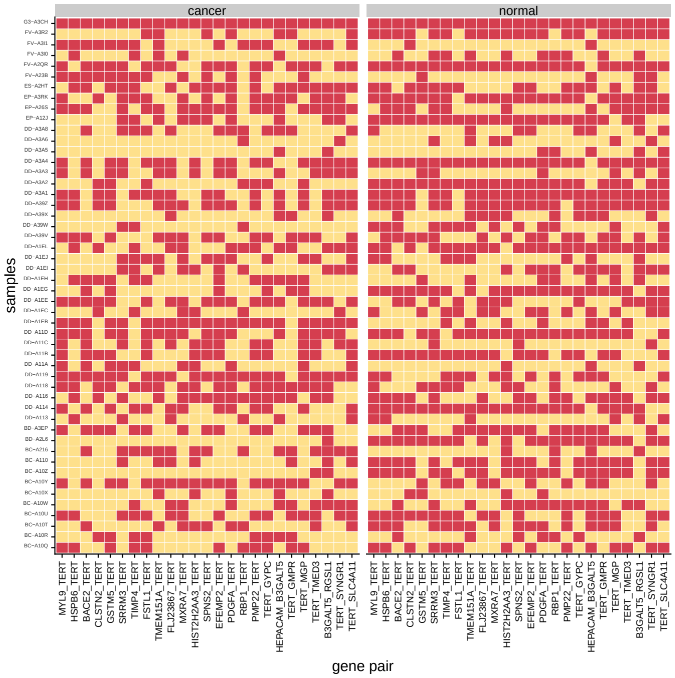

Fig. 4. Figure shows a heatmap representation of binary matrix constructed from the expression matrix of top differentially coexpressed gene pairs in LIHC data. Expression values of a gene pair showing the same pattern is indicated as '1' and showing a different pattern is indicated as '0' in the matrix. The columns representing differentially coexpressed gene pairs which show positive correlations normal stage while show negative correlation in cancer stage.

Supplementary figures for THCA data

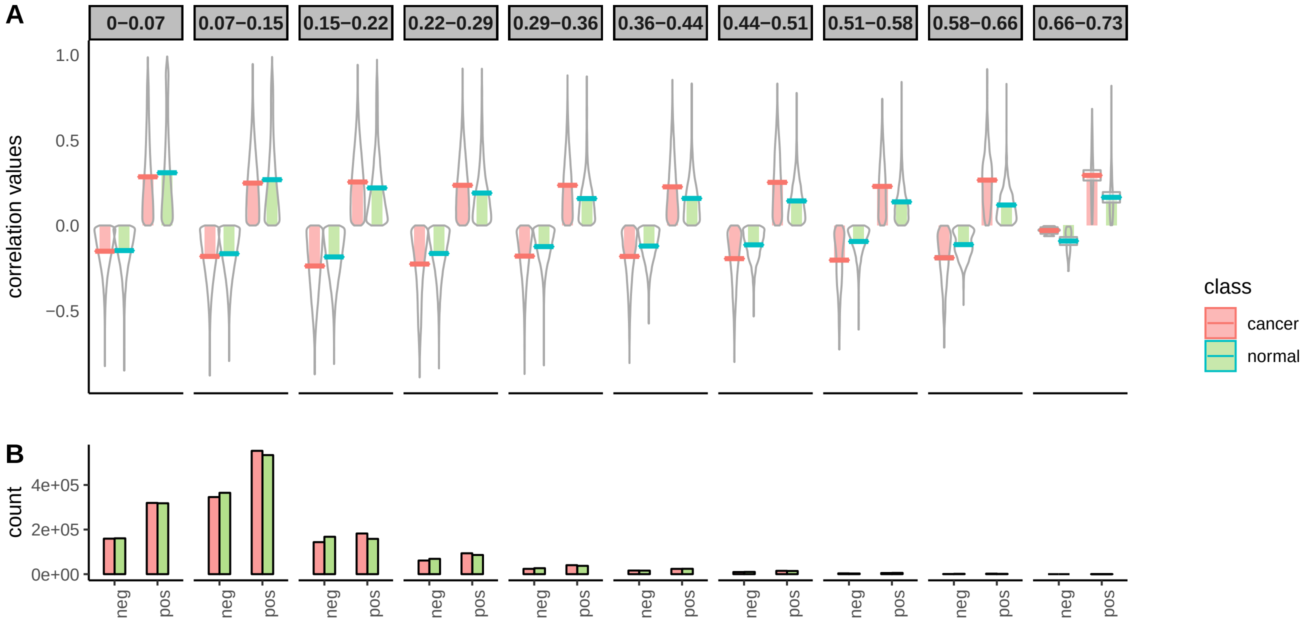

Fig. 1. Figure shows distribution of correlation values in normal and cancer samples of THCA data with the DC_Copula score. Panel-A shows the distribution for different DC_Copula scores. Here, 4 pirate plots are shown in each facet, two for positive and two for negative correlations. The violins in each facet represents the distribution of positive and negative correlations of gene pair in normal and cancer samples. Panel-B shows a bar plot representing the number of positive and negatively correlated gene pairs in normal and cancer samples in each facet

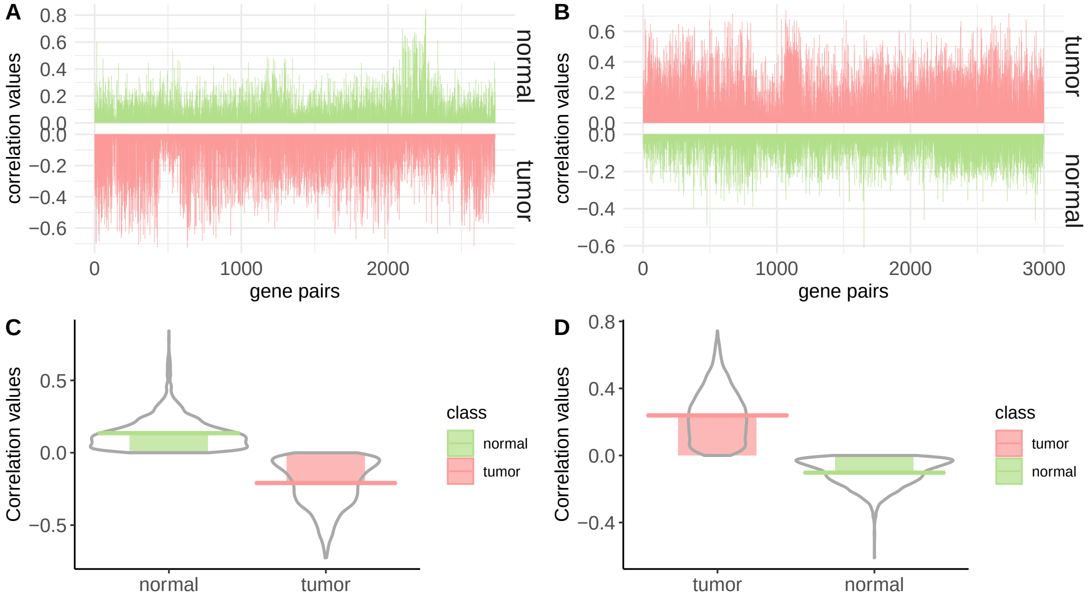

Fig. 2. Figure shows visualizations of gene pairs having DC_copula score greater than 0.56. Panel-A and Panel-B shows visualization of correlation values of gene pair having positive correlation in normal and negative correlation in tumor and vice-versa, respectively. Panel-C and Panel-D represents the distribution of correlation values according to panel-A and panel-B respectively.

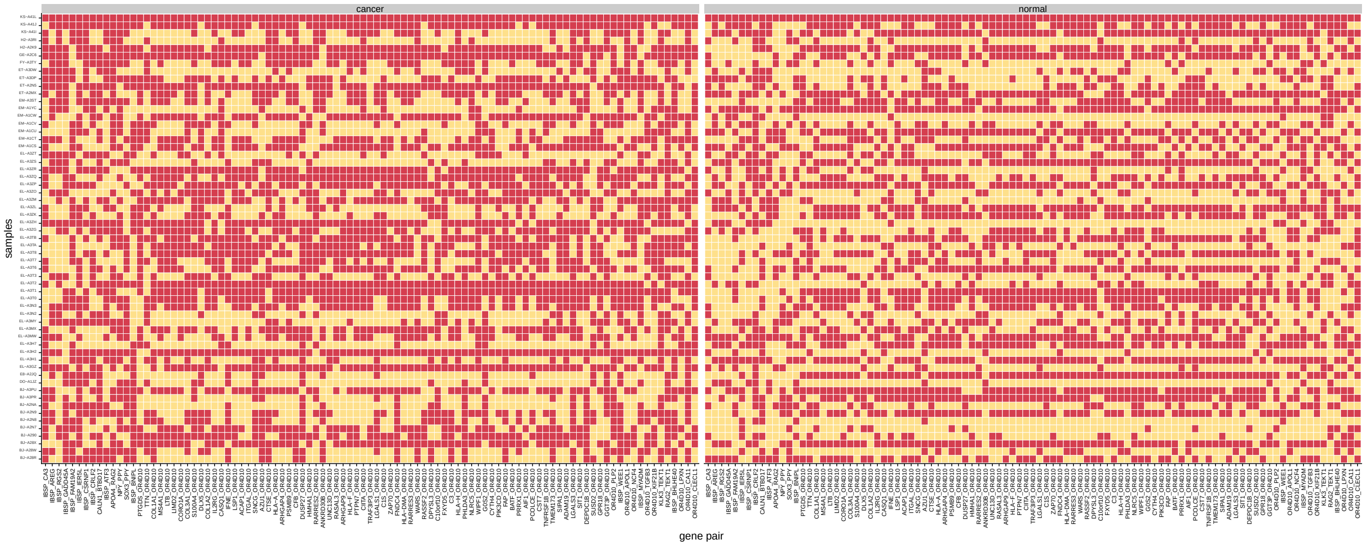

Fig. 3. Figure shows a heatmap representation of binary matrix constructed from the expression matrix of top differentially coexpressed gene pairs in THCA data. Expression values of a gene pair showing the same pattern is indicated as '1' and showing a different pattern is indicated as '0' in the matrix. The columns representing differentially coexpressed gene pairs which show positive correlations cancer stage while show negative correlation in normal stage.

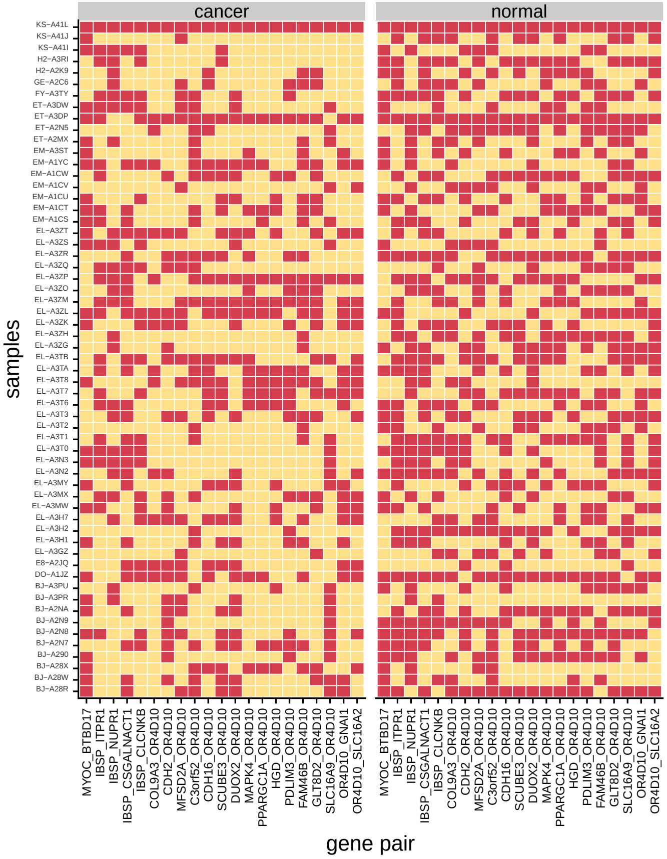

Fig. 4. Figure shows a heatmap representation of binary matrix constructed from the expression matrix of top differentially coexpressed gene pairs in THCA data. Expression values of a gene pair showing the same pattern is indicated as '1' and showing a different pattern is indicated as '0' in the matrix. The columns representing differentially coexpressed gene pairs which show positive correlations normal stage while show negative correlation in cancer stage.

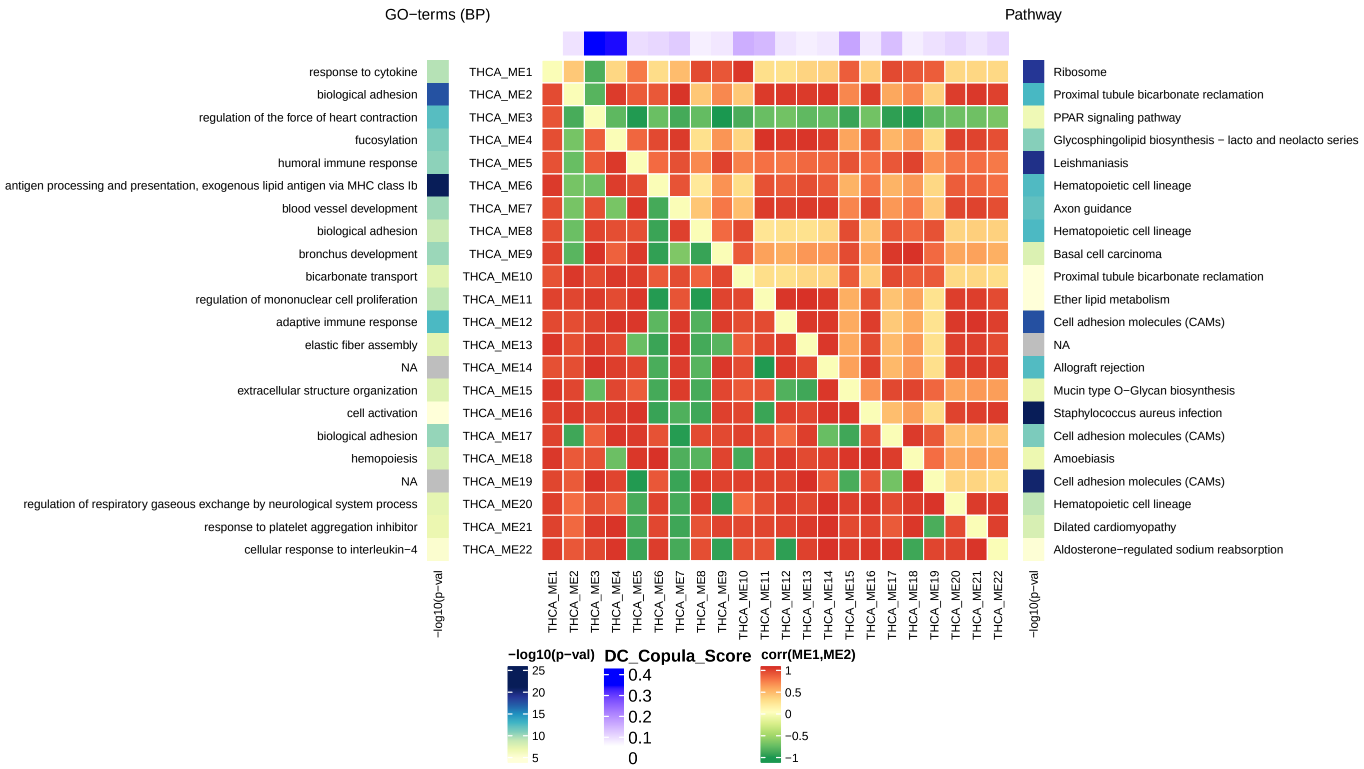

Fig. 5. Heatmap of differentially coexpressed modules. Here the heatmap is shown for module eigengenes.The upper triangular portion of the matrix represents correlations of module eigengenes in normal samples whereas lower triangular portion signifies the same for tumor samples. Left and right sidebar of the heatmap represents -log(p-value) of significantly enriched GO-terms and pathway, respectively. Upper annotation bar of the heatmap shows the DC_copula score of the module.

Supplementary figures for HNSC data

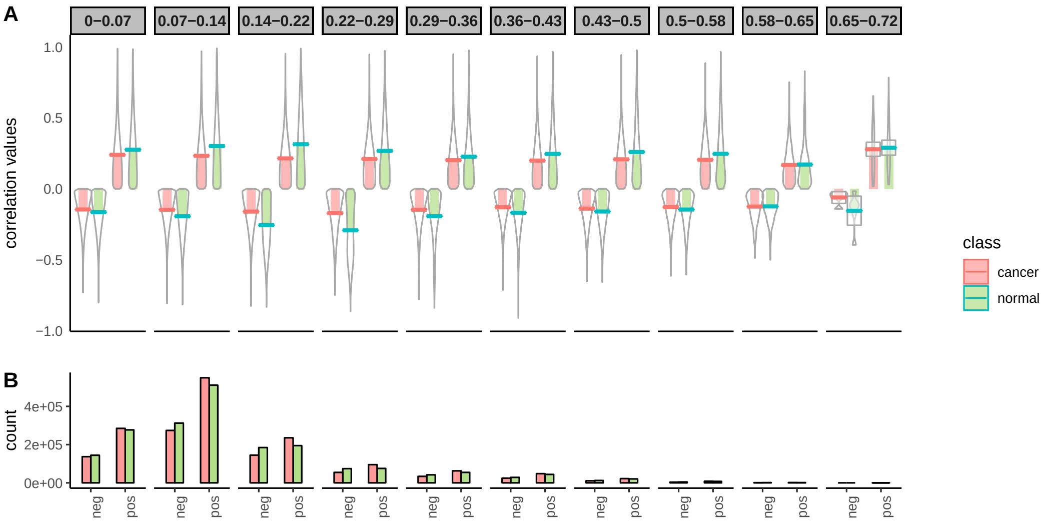

Fig. 1. Figure shows distribution of correlation values in normal and cancer samples of HNSC data with the DC_Copula score. Panel-A shows the distribution for different DC_Copula scores. Here, 4 pirate plots are shown in each facet, two for positive and two for negative correlations. The violins in each facet represents the distribution of positive and negative correlations of gene pair in normal and cancer samples. Panel-B shows a bar plot representing the number of positive and negatively correlated gene pairs in normal and cancer samples in each facet

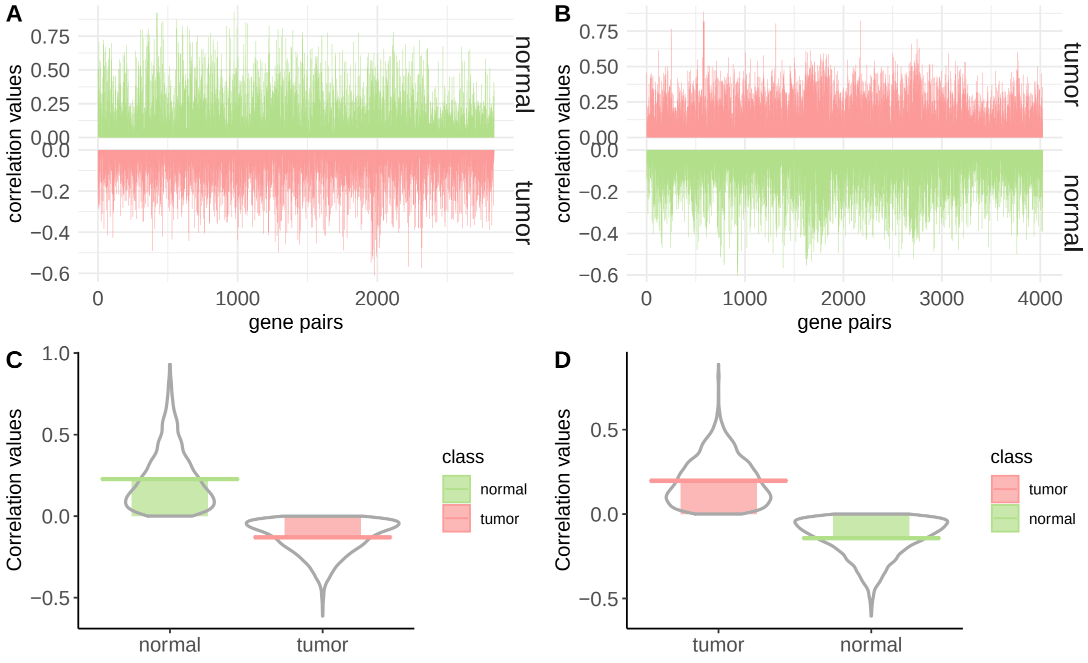

Fig. 2. Figure shows visualizations of gene pairs having DC_copula score greater than 0.56. Panel-A and Panel-B shows visualization of correlation values of gene pair having positive correlation in normal and negative correlation in tumor and vice-versa, respectively. Panel-C and Panel-D represents the distribution of correlation values according to panel-A and panel-B respectively.

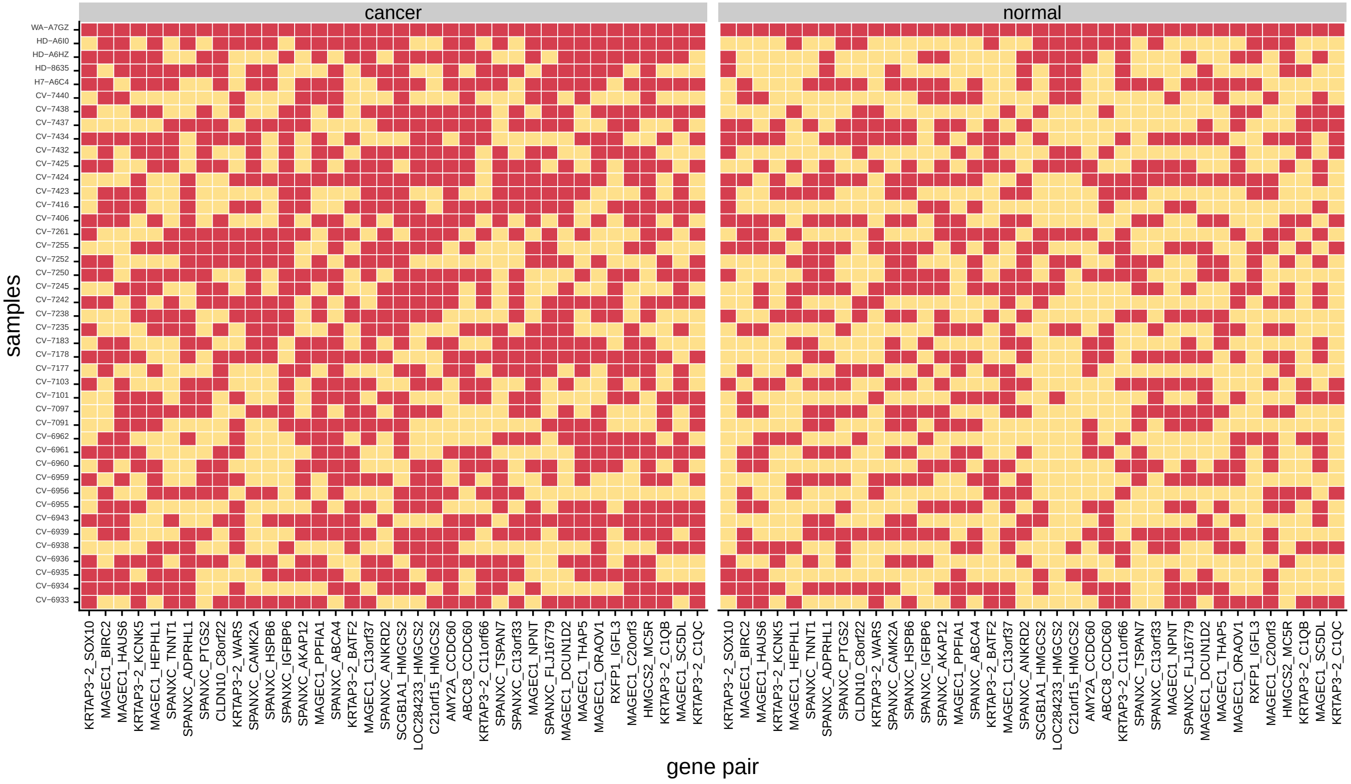

Fig. 3. Figure shows a heatmap representation of binary matrix constructed from the expression matrix of top differentially coexpressed gene pairs in HNSC data. Expression values of a gene pair showing the same pattern is indicated as ’1’ and showing a different pattern is indicated as ’0’ in the matrix. The columns representing differentially coexpressed gene pairs which show positive correlations cancer stage while show negative correlation in normal stage.

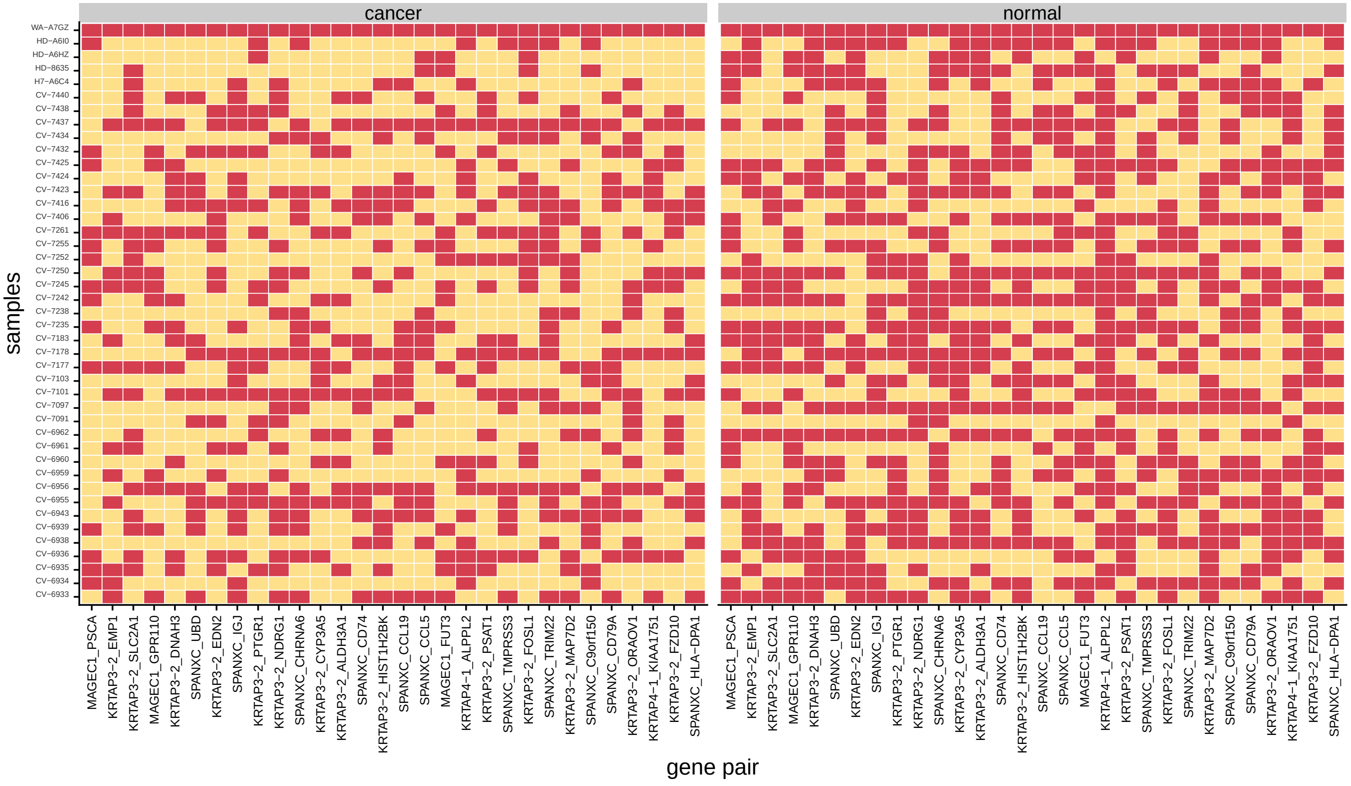

Fig. 4. Figure shows a heatmap representation of binary matrix constructed from the expression matrix of top differentially coexpressed gene pairs in HNSC data. Expression values of a gene pair showing the same pattern is indicated as ’1’ and showing a different pattern is indicated as ’0’ in the matrix. The columns representing differentially coexpressed gene pairs which show positive correlations normal stage while show negative correlation in cancer stage.
